# Silent rupture: The hidden danger of gastric perforation in paediatric blunt trauma: A case report

**DOI:** 10.6026/9732063002001313

**Published:** 2024-10-31

**Authors:** Ayushi Chotra, Vishal Bharadwaj, Deepak Goel, Kuldeep Sukhadeve, Priyanka Wankar, Rajkumar Kiratkar, Priyank Rajan, Mayur Wanjari

**Affiliations:** 1 Department of Paediatrics, Bristol Royal Hospital for Children, Bristol, United Kingdom; 2 Department of Paediatrics & Neonatology, KIMS-Kingsway Hospital, Nagpur, India; 3 Department of Paediatric Surgery, KIMS-Kingsway Hospital, Nagpur, India; 4 Paediatric Haematology Oncology & BMT, Bristol Royal Hospital for Children, Bristol, United Kingdom; 5 Department of Research, Datta Meghe Institute of Higher Education & Research (DMIHER), Sawangi, Maharashtra, India

**Keywords:** Paediatric, blunt abdominal trauma, gastric perforation, CT scan, surgical repair, systemic inflammatory response syndrome [SIRS], postoperative care

## Abstract

We present herein a case with the relatively uncommon presentation of isolated gastric perforation resulting from blunt trauma of the abdomen following a fall shortly after eating in a 5-year-old girl. The child experienced severe abdominal pain, tachycardia, hypotension, and shortness of breath. A physical examination revealed epigastric tenderness and abdominal distension. Initial X-ray was non-diagnostic, and a contrast-enhanced Computerised Tomography [CECT scan] demonstrated ascites and pneumoperitoneum that suggested hollow organ perforation. The emergency exploratory laparotomy revealed a 7 cm laceration on the stomach that was surgically repaired using two-layer closure. Following that, extensive peritoneal lavage occurred. In the postoperative period, the patient developed systemic inflammatory response syndrome [SIRS]. Hence, the PICU provided inotropic support and oxygen therapy. On the fourth day, she became significantly improved, and on the tenth day, we made her able to go home without any complications. The important points in the management of gastric perforation in paediatric blunt abdominal trauma are early suspicion, further imagination, and timely surgical intervention.

## Background:

One of the main causes of paediatric hospital admissions is blunt abdominal trauma, which accounts for 10% of all emergencies from trauma. By organs, it commonly occurs in splenic and hepatic injuries, as these organs are relatively larger in size within the abdominal cavity. Isolated gastric perforation, however, is very rare and occurs in only 0.02% to 1.7% of cases of paediatric blunt trauma [1, 2]. The muscular walls of the stomach, and its location-besides being a natural barrier to direct trauma-thus explain the rarity of such injuries. Its elasticity and position between other solid organs, including the liver and spleen, render this organ even less susceptible to injury. But even with such protection, the stomach is also one of the most dangerous when it is distended. Increased intra-gastric pressure from a full stomach can turn what otherwise could be commonplace compressive forces into catastrophes [3, 4]. Situations, which are examples of high-impact events, include road traffic accidents, falls from height, and sporting injuries that are usually attributed to the compressive forces that overwhelm the stomach and increase the chance of its rupture [5, 6]. The pliability of the abdominal wall and smaller size of children create more exposure to injury. Clinically, the early symptoms of isolated gastric perforation-such as abdominal pain, nausea, and vomitin-can are non-specific, closely mimicking more common injuries like splenic or liver trauma [7]. This often leads to diagnostic delays. While plain radiographs are typically the first-line imaging modality in trauma cases, they may fail to detect key signs of gastric perforation, such as free air in the abdomen [pneumoperitoneum] [7]. In contrast, computed tomography [CT] scans are far more sensitive and can provide a more definitive diagnosis by clearly identifying pneumoperitoneum, free fluid, and other associated injuries. Therefore, when there is a suspicion of gastric perforation, CT imaging is often the preferred diagnostic tool [8, 9 and 10].

## Case Presentation:

A 5-year-old girl was referred to the emergency department after falling during play; the injury occurred briefly after having a meal. She was brought in by her parents with complaints of acute abdominal pains, which had been worsening since the incident. She was admitted with suspicion of respiratory distress: tachypnoea with shallow and rapid respirations, tachycardia of 130 beats per minute, hypotension of 82/64 mmHg, with signs of shock. Physical examination demonstrated diffuse tenderness across the epigastric region, distended abdomen where there had been suspicion of visceral injury. These telltale signs, however, were belied by a plain abdominal X-ray examination, which usually would have documented the presence of free air-abdominal gas, or pneumoperitoneum, in the case of hollow organ perforation. A CECT scan was performed in light of the unremarkable X-ray film and the child's worsening state. It revealed moderate ascites and clear evidence of pneumoperitoneum, both of which were highly suggestive of hollow organ perforation.

Therefore, based on all these findings, it was decided to perform an emergency exploratory laparotomy to confirm the diagnosis and repair the injury. During the surgery, the surgeon noticed a 7-cm laceration on the stomach's anterior wall, which stretched from the fundus to the body. The peritoneal cavity was contaminated with gastric contents, a serious complication given the ensuing high risk of peritonitis and sepsis. Ryle's tube was identified protruding out of perforation into the peritoneal cavity. The surgical team proceeded with a two-layer gastric repair, using interrupted vicryl sutures to close the laceration. The surgical team also performed extended peritoneal lavage to remove potential stomach contaminants from the abdominal cavity, thereby lowering the risk of postoperative infection. Eventually, they transferred the child to the PICU for close observation. She developed features of SIRS within the first 24 hours-a serious complication that follows severe trauma or infection. High-flow nasal oxygen therapy managed her respiratory distress and inotropic support was required for hypotension. Despite these complications, by the fourth postoperative day, the patient gradually started to improve with stable vital signs and clinical condition improved. On the tenth day, he was discharged without any complications in stable conditions. Follow-up was scheduled to ensure further recovery and to observe the long-term results of the injury.

## Discussion:

Isolated gastric rupture following blunt abdominal trauma is exceedingly rare, but it can present significant diagnostic and therapeutic challenges. In paediatric patients, the condition is often underdiagnosed due to the non-specific presentation of symptoms such as abdominal pain, nausea, and vomiting. These symptoms can mimic more common injuries like splenic or liver trauma [11]. Early recognition of gastric perforation is critical, as delays in diagnosis can result in serious complications, including peritonitis, sepsis and even death. A high index of suspicion is necessary in paediatric patients presenting with abdominal trauma, especially if the trauma occurred after a meal, when the stomach is more vulnerable to rupture due to distention [12]. In this case, initial diagnostic imaging with plain radiography failed to identify pneumoperitoneum, which is often a telltale sign of hollow organ perforation [13]. Studies have shown that X-rays may not always reveal free air in the peritoneal cavity, especially in the early stages of perforation [14]. That is why many consider computed tomography [CT] the first choice in such cases. CT scans are very sensitive in identifying pneumoperitoneum, free fluid, and associated injuries and provide a much clearer view of the injury as well [15]. In the present case, the CT scan established the evidence of moderate ascites and pneumoperitoneum that necessitated emergency exploratory laparotomy [16]. Surgical intervention is the mainstay of treatment for gastric perforations. During the surgery, the surgeon is mandated to identify and close the perforation site using preferably two layers. In the current case, we closed a 7 cm laceration along the anterior wall of the stomach by two layers technique with interrupted sutures. We then did exhaustive peritoneal lavation to remove any gastric content which had spilled out and contaminated the abdominal cavity. Prevention of the development of more severe complications such as peritonitis that might culminate into septic shock and even multi-organ failure, which can be avoided only in case proper attention is paid to surgical repair of gastric perforations, makes surgical repair of gastric perforations important [17]. This patient developed systemic inflammatory response syndrome [SIRS] postoperatively, which remains one of the most common complications in cases of severe abdominal sepsis. SIRS is associated with excessive inflammatory response, leading to the sequelae of shock, organ failure and death if left unaggressive managed [18]. In this patient, she needed to be initiated on inotropic support and high-flow oxygen therapy for her stabilization. Early diagnosis and treatment of SIRS can impact patient outcomes because once the inflammatory cascade initiates it can deteriorate rather rapidly into more serious forms of sepsis [19]. With proper management in the ICU, the patient showed almost complete reversal of all his symptoms and was improving significantly by postoperative day four and such complex cases illustrate the imperative role of a multidisciplinary approach to manage them [20]. Though this remains a rare entity it was reportred as early as 1975 [21].

## Conclusion:

This case underscores the emergent and acute necessity for early suspicion and advanced imaging to diagnose rare gastric perforations. Symptoms may mimic more common injuries, and initial radiographs are often inconclusive. Thus, CT scans are required to make accurate diagnoses and ensure timely surgical intervention. Vigilant repair of the stomach and meticulous lavage of the peritoneum constitute a very vital activity in preventing deadly complications such as sepsis. SIRS should be the focus of postoperative management to facilitate recovery. The critical points in the patient affected with paediatric gastric perforation are proper early diagnosis and rapid surgery followed by an intensive care measures that ensure effective recovery with minimal complications.
